# In Search of a Global Distress Measurement Instrument for Perinatal Use: Testing Depression Anxiety Stress Scales Short Forms with Swedish Pregnant and Postpartum Women

**DOI:** 10.3390/healthcare14121636

**Published:** 2026-06-10

**Authors:** Birgitta Kerstis, Peter Jönsson, Alyx Taylor, Kent W. Nilsson, Björn Hofvander, Christine Rubertsson, Sara Lindeberg

**Affiliations:** 1 Department of Health Sciences, Innovation and Design, Mälardalen University, 721 23 Västerås, Sweden; kent.nilsson@regionvastmanland.se; 2 Department of Psychology, Faculty of Education, Kristianstad University, 291 88 Kristianstad, Sweden; peter.jonsson@hkr.se; 3 School of Health and Rehabilitation Sciences, Health Sciences University, Bournemouth BH5 2DF, UK; alyx.taylor@hsu.ac.uk; 4 Center for Clinical Research, Central Hospital of Västerås, Uppsala University, 753 10 Uppsala, Sweden; 5 Department of Clinical Sciences Lund, Faculty of Medicine, Lund University, 221 00 Lund, Sweden; bjorn.hofvander@med.lu.se (B.H.); sara.lindeberg@med.lu.se (S.L.); 6 Department of Forensic Psychiatry, Skåne University Hospital, 205 02 Malmö, Sweden; 7 Department of Health Sciences, Faculty of Medicine, Lund University, 221 00 Lund, Sweden; christine.rubertsson@med.lu.se; 8 Department of Psychiatry, Skåne University Hospital, 251 87 Helsingborg, Sweden

**Keywords:** anxiety, depression, third pregnancy trimester, postpartum period, psychological distress, psychometrics, stress

## Abstract

**Background/Objectives**: Valid and time-efficient measurement instruments for the assessment of perinatal distress beyond depressive symptoms are yet to be determined. The main objective was to analyse the psychometric measurement properties of the Depression Anxiety Stress Scales (DASS) short forms in Swedish pregnant women during the third trimester. The secondary objective was to analyse the measurement properties of DASS short forms for the postnatal period. **Methods**: Data from the Scania Birth Cohort study including 78 women followed prospectively from the third pregnancy trimester to one year postpartum were used. The DASS-21, DASS-12, DASS-9 (two versions), and the 12-item Mini-DASS were analysed using confirmatory factor analysis (CFA), inter-item analysis, and Spearman’s rho subscale cross-correlations. Postnatal analysis at infant ages 1, 6, and 12 months was performed using CFA and inter-item analysis. **Results**: When used with third-trimester pregnant women, the DASS-9 version 1 and the Mini-DASS exhibited overall acceptable psychometric properties in terms of internal consistency (McDonald’s ω ≥ 0.77) and structural and discriminant validity (e.g., CFI > 0.90 and SRMR < 0.08 for all DASS-9 two- and three-factor models; and CFI > 0.95 and SRMR < 0.08 for one-factor models of the Mini-DASS subscales - including a modified anxiety scale - and for the Mini-DASS depression and anxiety two-factor model). Support for these DASS short forms postpartum was also indicated. **Conclusions**: Although preliminary, the current results support the DASS-9 and the Mini-DASS as parsimonious tools for the assessment of perinatal distress and its subtypes. Further validation in the perinatal context is warranted.

## 1. Introduction

Perinatal distress is defined as one or more states of parental depression or depressive symptoms, anxiety, and stress between conception and one year postpartum [1]. A substantial proportion of women worldwide experience psychological distress during the perinatal period [1,2,3,4,5,6,7]. For the broader construct of perinatal distress, including symptoms of anxiety, depression, and stress, prevalence estimates have ranged between 20 and 30% [5]. For perinatal anxiety and depressive symptoms, respectively, studies performed during the COVID-19 pandemic indicated that prevalences increased [6]. Perinatal maternal health is well established to have long-term implications for the offspring, such as impacting the child’s sensitivity to maternal signals, child sleeping patterns, and motor, cognitive, language, socio-emotional, and behavioural development [8,9]. Population-level support systems for promotion, screening, and targeted interventions to facilitate the transition to parenthood would therefore be desirable, with expected benefits for the parents and their children.

To date, most of the research on perinatal distress has been conducted on postnatal maternal depressive symptoms [8,10]. Perinatal distress has frequently been assessed using instruments developed for assessing mental health constructs in the general population [11,12]. It has been pointed out that several “somatic” distress symptoms, such as fatigue and a change in appetite, often included in general mental health constructs, can be regarded as normally occurring somatic manifestations during pregnancy and after birth and therefore may not be suitable for the perinatal context [2,13,14]. However, a global distress measurement instrument that is valid for different types of populations, including general and clinical as well as perinatal populations, would entail benefits, for example, through the facilitation of comparisons of distress levels between these populations. Therefore, it is important that the psychometric properties of mental health instruments are tested when introducing them into the perinatal context.

The Depression Anxiety Stress Scales (DASS) includes three self-reported scales designed to measure the emotional states of depression, anxiety, and stress in adults [15]. The 21-item DASS (DASS-21) [16] is a shorter version of the original 42-item DASS, translated into more than 50 languages [17,18] and exhibits psychometric robustness in general and clinical populations across different countries [18,19]. The DASS-21 has frequently been considered useful for capturing the broader concept of perinatal distress, including anxiety and stress besides depression [12,20,21]. Only one study [22] has assessed its psychometric properties for pregnant women. For the postpartum period, its validity has been indicated for Swedish mothers and fathers [23] and for Czech mothers [17].

From the DASS-21, even shorter versions have been derived [24,25,26,27,28]. Motives for developing these shorter DASS forms have included the demands for efficient questionnaires that reduce the burden on respondents, which is a frequent request in perinatal screening contexts [10], while also maintaining or even enhancing psychometric validity [24]. None of the existing shorter versions derived from the DASS-21 have been validated in a perinatal population.

The main study aim was to evaluate the psychometric measurement properties of the DASS-21 and four of its short forms in Swedish women during their third trimester of pregnancy. Only DASS short forms with balanced subscales (i.e., an equal number of items for depression, anxiety, and stress in the respective forms) were included in the study to optimise subscale analysis comparability. Accordingly, the DASS-21, DASS-12, DASS-9 (two different versions), and the 12-item Mini-DASS were scrutinised and analysed. The focus of the analysis was on the separate predefined subscales and their relations with each other (internal consistency, structural validity, discriminant validity). A secondary aim was to validate a selection of the DASS versions with postnatal maternal data collected when the infants were 1, 6, and 12 months old.

## 2. Materials and Methods

### 2.1. Recruitment of Participants and Data Collection

This study used survey data from the first four waves of the Scania Birth Cohort—Health development in a life-course perspective pilot study [23,29], with the first wave during the third trimester and the following three waves at 1, 6 and 12 months postpartum. Seventy-eight women who attended an antenatal clinic at a primary healthcare centre in Scania, the most Southern region of Sweden, were recruited between February 2019 and June 2021, during a routine visit in gestation week 28. Eligibility criteria were attendance at the clinic, singleton pregnancy, and sufficient proficiency in Swedish, i.e., not needing an interpreter at healthcare visits. Study participants who did not reach term (pregnancy week 37) were excluded from the study. All women received a questionnaire in week 28 and completed it before giving birth, i.e., during the third trimester. After birth, the children were enrolled in postnatal care following the standard no-cost national programme for Swedish Child Health Services (CHS). Questionnaire data were collected from the mothers at the CHS centre between May 2019 and June 2021, during visits when the infants were 1 month (*n* = 62), 6 months (*n* = 57), and 12 months (*n* = 36) old. Due to the prolonged restrictions from the COVID-19 pandemic, participant recruitment and CHS centre data collection were stopped prematurely in June 2021, which is the main reason for the declining numbers of participants during the postnatal period [23].

### 2.2. The Depression Anxiety Stress Scales (DASS) Short Forms

The DASS-21 [16] is a shortened version of the original 42-item DASS [30]. Each DASS-21 subscale—depression (e.g., “I couldn’t seem to experience any positive feeling at all”), anxiety (e.g., “I was aware of dryness of my mouth”), and stress (e.g., “I found it hard to wind down”)—includes seven items scored on a four-point scale [31]. The Swedish version of the DASS-21 [32] was utilised. When scrutinising the DASS-21 for items that could reflect “somatic” symptoms, it was found that only the anxiety scale contained such items. Specifically, four out of the seven DASS-21 anxiety scale items could be perceived as somatic symptoms, i.e., “I was aware of dryness of my mouth”, “I experienced breathing difficulty (e.g., excessively rapid breathing, breathlessness in the absence of physical exertion)”, “I experienced trembling (e.g., in the hands)”, and “I was aware of the action of my heart in the absence of physical exertion (e.g., sense of heart rate increase, heart missing a beat)”. The other three DASS-21 anxiety scale items are “I was worried about situations in which I might panic and make a fool of myself”, “I felt I was close to panic”, and “I felt scared without any good reason”.

The DASS-12 [26,27], the 12-item Mini-DASS [24], and two different DASS-9 versions [25,28] are shorter versions derived from the DASS-21, including either 12 or 9 items equally distributed between their depression, anxiety, and stress subscales. The DASS-12 [26,27] anxiety subscale contains all four “somatic” anxiety items described above. In the two DASS-9 versions, each 3-item anxiety scale contains one “somatic” symptom item, i.e., “I experienced trembling” [25] or “I experienced breathing difficulty” [28], respectively. The Mini-DASS [24] 4-item anxiety scale contains two “somatic” symptom items, i.e., “I experienced trembling” and “I was aware of the action of my heart in the absence of physical exertion”. In the original validation process of the Mini-DASS [24], it was found that the anxiety item “I felt I was close to panic” was somewhat more informative than “I experienced trembling”, whereas the latter was more discriminative. Inspired by these former results, a slightly modified Mini-DASS, substituting the anxiety item “I experienced trembling” with “I felt I was close to panic”, was also tested in the current study.

### 2.3. Statistical Analysis

In confirmatory factor analysis (CFA), the following factor models of the DASS-21 and its short forms were tested separately for each ante- and postnatal timepoint, i.e., one-factor models containing items from each separate DASS subscale (depression, anxiety, and stress); one-factor models including all the respective total scale items; and, two- and three-factor models including two or three of the subscale factors of depression, anxiety, and stress. CFA model fit was assessed using the following criteria [33]: models with chi-square *p*-values greater than 0.05, Comparative Fit Index (CFI) and Tucker–Lewis Index (TLI) values greater than 0.95, Standardised Root Mean Square Residual (SRMR) values less than 0.08, and Root Mean Square Error of Approximation (RMSEA) values less than 0.06 were adopted as indicating good model fit, while CFI values between 0.90 and 0.95 in combination with SRMR less than 0.08 were reported to indicate adequate fit. Confirmatory factor analysis (CFA) was performed on ordinal data using Maximum Likelihood (ML) with Full Information Maximum Likelihood (FIML) for missing data handling, and with factor variances set to 1. Internal consistency was assessed through inter-item analysis using the McDonald’s omega (ω) coefficient. Spearman’s rank correlation method (rho) was used to analyse cross-correlations between DASS subscale scores during the third trimester of pregnancy. Descriptive statistics and correlations were performed using IBM SPSS Statistics (version 29.0; IBM Corp., Armonk, NY, USA). For CFA and inter-item analysis, jamovi (version 2.6.44) retrieved from www.jamovi.org was used.

## 3. Results

### 3.1. Description of the Sample

The descriptive characteristics of the pregnant cohort are presented in Table 1.

### 3.2. Psychometric Properties of the DASS Short Forms During the Third Trimester

#### 3.2.1. Confirmatory Factor Analysis Results

The two DASS-9 subscale one-factor models including three items each did not converge in CFA, i.e., they did not reach a stable solution. The Mini-DASS exhibited adequate fit for all three subscales, with the best fit for the Mini-DASS anxiety scale (*x*^2^ = 4.08, *p* = 0.192; CFI = 0.988 and TLI = 0.963; SRMR = 0.039 and RMSEA = 0.091). The modified Mini-DASS anxiety subscale one-factor model fulfilled all the criteria for good model fit (*x*^2^ = 2.22, *p* = 0.330; CFI = 0.999; TLI = 0.996; SRMR = 0.012; RMSEA = 0.037). The total scale one-factor models produced poorer model fit in all instances as compared to their respective three-factor models, indicating that the three-factor models better explained the variance than the total scale one-factor models did. No three-factor model exhibited good model fit, and only the DASS-9 version 1 and DASS-12 three-factor models had adequate fit. Neither the DASS-21, DASS-12, nor DASS-9 version 2 one-, two-, or three-factor model fulfilled the criteria for good model fit (Table 2).

Out of the DASS two-factor models, the best fit indices were produced by the DASS-9 version 1 and Mini-DASS two-factor models including the depression and anxiety factors (x^2^ = 12.6, *p* = 0.127; CFI = 0.978; TLI = 0.958; SRMR = 0.047; RMSEA = 0.086 and *x*^2^ = 25.3, *p* = 0.150; CFI = 0.983; TLI = 0.975; SRMR = 0.0421; RMSEA = 0.065, respectively), the latter fulfilling all the criteria for good model fit. The DASS-9 version 1 exhibited adequate fit for the other two two-factor models, and the Mini-DASS had adequate fit for the depression and stress two-factor model (Table 2).

#### 3.2.2. Internal Consistency

The McDonald’s ω internal reliability values for the DASS short-form subscales were all ≥0.70, indicating good internal consistency. The lowest McDonald’s ω was observed for the DASS-12 anxiety subscale (ω = 0.70) (Table 2).

#### 3.2.3. Cross-Correlations Between DASS Short Form Subscale Scores

Cross-correlations between the respective DASS short form subscale scores were low-to-moderate for the depression and anxiety scores, ranging between *r_s_* = 0.27, *p* = 0.009 (DASS-12) and *r_s_* = 0.55, *p* < 0.001 (DASS-9 version 1), as well as for the anxiety and stress scores, ranging between *r_s_* = 0.37, *p* < 0.001 (DASS-12) and *r_s_* = 0.56, *p* < 0.001 (modified Mini-DASS). Correlations were moderate-to-strong for the DASS depression and stress scale scores, ranging between *r_s_* = 0.52, *p* < 0.001 (DASS-9 version 2) and *r_s_* = 0.76, *p* < 0.001 (DASS-21) (Table 3).

The DASS-21-derived short form depression and anxiety scores generally correlated strongly with the DASS-21 depression and anxiety scores. The DASS-21-derived short form stress scores correlated very strongly with the DASS-21 stress score in all cases, ranging between *r_s_* = 0.86, *p* < 0.001 and *r_s_* = 0.97, *p* < 0.001 (Table 3).

### 3.3. Psychometric Properties of DASS Short Forms Using Maternal Postnatal Data

Based on the antenatal results, the DASS-9 version 1 and the Mini-DASS, including its modified form, were selected for analysis with maternal postnatal data at infant ages 1, 6 and 12 months. The DASS-9 version 1 subscale one-factor models did not converge in CFA. The Mini-DASS subscale one-factor models, including its herein modified anxiety scale, exhibited good or adequate CFA model fit at all three postnatal timepoints, except for the Mini-DASS depression scale at postnatal month 12. McDonald’s ω was ≥ 0.70 at all timepoints, except for the DASS-9 depression scale at postnatal month 12 (ω = 0.62) (Table 4).

The DASS-9 version 1 two-factor models at postnatal month 1 including depression and anxiety, and anxiety and stress, respectively, produced adequate fit (*x*^2^ = 23.4, *p* = 0.003; CFI = 0.916; TLI = 0.842; SRMR = 0.042; RMSEA = 0.19 and *x*^2^ = 17.9, *p* = 0.022; CFI = 0.928; TLI = 0.864; SRMR = 0.079; RMSEA = 0.14, respectively). The DASS-9 version 1 two-factor model including depression and stress at postnatal month 6 fulfilled the criteria for good model fit (*x*^2^ = 8.07, *p* = 0.427; CFI = 0.999; TLI = 0.999; SRMR = 0.050; RMSEA = 0.012). The other two-factor models of the DASS-9, as well as its total scale one-factor model and its three-factor model, produced poor model fit. No Mini-DASS two- or three-factor models or total scale one-factor model produced adequate fit.

## 4. Discussion

The present study aimed to evaluate the psychometric properties of the DASS-21 and four of its short forms in a perinatal context, focusing on third-trimester pregnancy and extending the analysis into the first year postpartum. Overall, the third-trimester pregnancy findings suggested comparatively more favourable psychometric properties of the DASS-9 version 1 [25] and the Mini-DASS [24] in terms of structural validity, internal consistency, and discriminant validity as compared to the other forms evaluated here. Support for the factor structure and internal consistency of these two DASS short forms was also found for postpartum mothers.

To our knowledge, this is the first study to perform CFA on either the DASS-21 or any of its short forms in pregnant women. It is also the first to analyse DASS-21-derived short forms in postpartum mothers. Therefore, comparisons with previous studies cannot be made in these regards. One psychometric study [22] of Portuguese versions of the DASS-21 used antenatally found satisfactory internal consistencies for all three subscales, in agreement with our findings.

Notably, the two Mini-DASS [24] anxiety scale versions examined in this study, including one and two “somatic” anxiety items, respectively, performed better than the DASS-12, which includes four “somatic” items in its anxiety scale. In the pregnant sample studied here, the modified Mini-DASS anxiety scale including only one “somatic” anxiety symptom had an excellent model fit in the CFA, and its internal consistency was superior to that of the original Mini-DASS anxiety scale. However, its discriminant validity against depression was found to be inferior to that of the original Mini-DASS, seemingly in agreement with the findings presented by Monteiro et al. [24]. Moreover, the current results suggest that these two anxiety scales perform equally well during the postnatal period. 

The three-dimensional structures were supported as compared to their respective unidimensional total scale structures, although no DASS version studied here fulfilled the criteria for good CFA three-factor model fit. These results are in line with those presented for the original DASS-42 [15] and may be explained by the well-known cross-correlations between the DASS subscales [15,16]. Considering that the original quest leading to the development of the DASS was the widespread cumbersome overlap between depression and anxiety in various self-report scales [15], it is satisfying that the results presented here indicate that the DASS-9 version 1 and the Mini-DASS both discriminate well between these two conditions in third-trimester pregnant women, while also discriminating adequately between depression and stress. Interestingly, the highest cross-correlations observed here were for the various forms of DASS depression and stress scales. This pattern contrasts with that initially reported for the DASS-42 and DASS-21, for which the highest correlations were seen between the anxiety and stress scales [15,16]. In addition, the correlation pattern found here does not clearly align with correlation patterns observed for postpartum mothers using the DASS-21 [17,23]. Further research is needed to clarify a potential overlap between depressive symptoms and stress in pregnant women.

The present findings indicate that shorter DASS versions may retain acceptable psychometric properties while reducing respondent burden, which is particularly relevant in perinatal contexts characterised by screening fatigue and competing demands. The DASS also provides a general dimensional framework that may capture depression, anxiety and stress simultaneously across populations. It is, however, not suggested that the DASS short forms analysed here would be suitable in all possible situations. When a more context-specific measurement is needed, there are several validated measures to choose from that are designed specifically for certain perinatal populations and that are longer and may cover additional domains. Examples of these are the Edinburgh Postnatal Depression Scale (EPDS) [34], the Perinatal Anxiety Screening Scale (PASS) [35] (yielding a four-factor structure covering distinct anxiety domains), the Tilburg Pregnancy Distress Scale [36] (measuring distress and perceived partner involvement), and the Prenatal Distress Questionnaire (PDQ) [37] (focusing on pregnancy-specific worries). Choosing a distress measurement instrument usually involves a trade-off between, for example, specificity, generalizability and feasibility, and the DASS-9 and the Mini-DASS may prove to facilitate a broad applicability and comparability across populations.

A strength of this study is its, in this context, unique or rare longitudinal design incorporating repeated assessments from late pregnancy to 12 months postpartum, thereby constituting a novel contribution to the DASS validation literature. The consistency of the results across timepoints strengthens their interpretation. A limitation of the study is the small sample sizes, warranting caution when interpreting the CFA results, particularly concerning the DASS-21, whose larger number of items compared to the other included DASS versions requires more participants for stable model estimation [38]. The drop in the numbers of study participants across timepoints due to administrative issues connected to the COVID-19 pandemic accentuated this limitation concerning the postnatal data. Measurement invariance assessments were not performed and are recommended for future studies, including, for example, assessments of differential item functioning (DIF) of the “somatic” items across trimesters and postpartum periods to evaluate their suitability for the perinatal context. Further, the study sample consisted of Swedish-speaking women recruited from a single healthcare setting, which may limit external validity. No external clinical validation was performed, and further validation, including, for example, criterion validity and the specificity of the included “somatic” symptoms to anxiety in perinatal populations, is warranted.

## 5. Conclusions

The study provides evidence for the psychometric performance of DASS short forms in a perinatal context, demonstrating support for the DASS-9 [25] and the Mini-DASS [24]. They may be useful as parsimonious and time-efficient tools for mental health screening and research during the perinatal period. Continued validation in perinatal populations is desired.

## Figures and Tables

**Table 1:** Demographic background characteristics and 21-item Depression Anxiety Stress Scales (DASS-21) scores of the third-trimester pregnant women (*N* = 78).

Characteristics		*N* = 78
**Age mean (SD)**		31.0 (4.1)
**Highest education *n* (%)**		
Compulsory school (9 years)		3 (3.9)
Senior high school		24 (30.8)
University (≤3 years)		23 (29.9)
University (>3 years)		27 (35.1)
Post-graduate education		0
**Occupation status *n* (%)**		
Parental leave, employed		21 (26.9)
Parental leave, unemployed		5 (6.4)
Student		2 (2.6)
Employed		46 (59.0)
Self-employed		1 (1.3)
On sick leave		1 (1.3)
Unemployed		2 (2.6)
**Cohabitation Status *n* (%)**		
Living with the other parent		71 (91.0)
Living alone		5 (6.4)
Living with their own parent/-s		2 (2.6)
**Region of birth *n* (%)**		
Sweden		70 (89.7)
Scandinavia (not including Sweden)		1 (1.3)
Europe (not including Scandinavia)		4 (5.1)
Outside of Europe		3 (3.8)
**DASS-21 subscale central tendencies**	**Mean (SD)**	Median (IQR) *
DASS-21 depression	2.39 (3.31)	1 (3)
DASS-21 anxiety	1.99 (2.84)	1 (2)
DASS-21 stress	4.03 (3.47)	3 (7)

* IQR = Interquartile range.

**Table 2:** Fit indices for confirmatory factor analysis (CFA) models of the Depression Anxiety Stress Scales (DASS)-21 and its short forms and scale internal reliability values (McDonald’s ω), analysed with data from third-trimester pregnant women (*N* = 78).

	Models	Third-Trimester Pregnancy CFA Model Fit Indices							
		*χ* ^2^	*df*	*p*	CFI	TLI	SRMR	RMSEA	ω
**DASS-21** ^1^ ***One***-, ***Two***- and ***Three***-factor models:									
(1)	***One*** (depression, 7 items)	31.7	14	0.004	**0.952**	*0.928*	**0.040**	0.127	0.92
(2)	***One*** (anxiety, 7 items)	41.5	14	<0.001	0.874	0.811	0.077	0.159	0.84
(3)	***One*** (stress, 7 items)	64.8	14	<0.001	0.769	0.653	0.099	0.216	0.84
(4)	***One*** (total scale, 21 items)	516	189	<0.001	0.700	0.667	0.094	0.149	0.94
(5)	***Two*** (depression, anxiety)	175	76	<0.001	0.861	0.833	0.077	0.104	
(6)	***Two*** (depression, stress)	219	76	<0.001	0.798	0.759	0.085	0.155	
(7)	***Two*** (anxiety, stress)	180	76	<0.001	0.796	0.755	0.087	0.132	
(8)	***Three*** (depression, anxiety, stress)	442	186	<0.001	0.765	0.735	0.088	0.133	
**DASS-12** ^2^ ***One****-, **Two***- and ***Three***-factor models:									
(1)	***One*** (depression, 4 items)	5.86	2	**0.054**	**0.976**	*0.927*	**0.030**	0.157	0.90
(2)	***One*** (anxiety, 4 items)	7.06	2	0.029	*0.910*	0.729	**0.051**	0.180	0.70
(3)	***One*** (stress, 4 items)	12.9	2	0.002	0.868	0.605	0.076	0.265	0.75
(4)	***One*** (total scale, 12 items)	135	54	<0.001	0.788	0.741	0.090	0.138	0.88
(5)	***Two*** (depression, anxiety)	36.5	19	0.009	*0.928*	0.894	**0.071**	0.109	
(6)	***Two*** (depression, stress)	45.9	19	<0.001	*0.906*	0.862	**0.066**	0.135	
(7)	***Two*** (anxiety, stress)	45.0	19	<0.001	0.846	0.772	0.086	0.132	
(8)	***Three*** (depression, anxiety, stress)	64.2	40	0.009	*0.929*	*0.902*	**0.070**	0.088	
**DASS-9** v.1 ^3^ ***One****-, **Two***- and ***Three***-factor models:									
(1)	***One*** (depression, 3 items)	- ^7^							0.78
(2)	***One*** (anxiety, 3 items)	- ^7^							0.79
(3)	***One*** (stress, 3 items)	- ^7^							0.77
(4)	***One*** (total scale, 9 items)	73.5	27	<0.001	0.857	0.810	0.066	0.149	0.89
(5)	***Two*** (depression, anxiety)	12.6	8	**0.127**	**0.978**	**0.958**	**0.047**	0.086	
(6)	***Two*** (depression, stress)	25.2	8	0.001	*0.902*	0.816	**0.063**	0.166	
(7)	***Two*** (anxiety, stress)	14.3	8	**0.075**	**0.962**	*0.930*	**0.042**	0.100	
(8)	***Three*** (depression, anxiety, stress)	50.8	24	0.001	*0.918*	0.877	**0.062**	0.120	
**DASS-9** v.2 ^4^ ***One****-, **Two***- and ***Three***-factor models:									
(1)	***One*** (depression, 3 items)	- ^7^							0.88
(2)	***One*** (anxiety, 3 items)	- ^7^							0.76
(3)	***One*** (stress, 3 items)	- ^7^							0.71
(4)	***One*** (total scale, 9 items)	82.1	27	<0.001	0.853	0.804	0.083	0.162	0.88
(5)	***Two*** (depression, anxiety)	22.4	8	0.004	**0.951**	0.907	**0.043**	0.152	
(6)	***Two*** (depression, stress)	43.2	9	<0.001	0.756	0.594	0.096	0.221	
(7)	***Two*** (anxiety, stress)	13.3	8	**0.101**	**0.973**	*0.949*	**0.066**	0.093	
(8)	***Three*** (depression, anxiety, stress)	58.9	24	0.001	0.907	0.860	0.081	0.137	
**Mini-DASS** ^5^ ***One****-, **Two***- and ***Three***-factor models:									
(1)	***One*** (depression, 4 items)	8.11	2	0.017	**0.969**	*0.908*	**0.028**	0.198	0.90
(2)	***One*** (anxiety, 4 items)	3.30	2	**0.192**	**0.988**	**0.963**	**0.039**	0.091	0.81
(3)	***One*** (stress, 4 items)	4.08	2	**0.130**	**0.979**	*0.938*	**0.033**	0.116	0.81
(4)	***One*** (total scale, 12 items)	180	54	<0.001	0.770	0.719	0.092	0.173	0.92
(5)	***Two*** (depression, anxiety)	25.3	19	**0.150**	**0.983**	**0.975**	**0.042**	**0.065**	
(6)	***Two*** (depression, stress)	47.3	19	<0.001	*0.919*	0.880	**0.060**	0.138	
(7)	***Two*** (anxiety, stress)	46.3	19	<0.001	0.892	0.841	0.086	0.136	
(8)	***Three*** (depression, anxiety, stress)	113	51	<0.001	0.887	0.854	**0.077**	0.125	
**Modified Mini-DASS** ^6^ ***One***-, ***Two***- and ***Three***-factor models:									
(1)	***One*** (depression, 4 items)	8.11	2	0.017	**0.969**	0.908	**0.028**	0.198	0.90
(2)	***One*** (anxiety, 4 items)	2.22	2	**0.330**	**0.999**	**0.996**	**0.019**	**0.037**	0.86
(3)	***One*** (stress, 4 items)	4.08	2	**0.130**	**0.979**	**0.938**	**0.033**	0.116	0.81
(4)	***One*** (total scale, 12 items)	183	54	<0.001	0.791	0.744	0.090	0.175	0.93
(5)	***Two*** (depression, anxiety)	47.2	19	<0.001	*0.937*	*0.906*	**0.048**	0.138	
(6)	***Two*** (depression, stress)	47.3	19	<0.001	*0.919*	0.880	**0.060**	0.138	
(7)	***Two*** (anxiety, stress)	50.9	19	<0.001	0.893	0.842	**0.074**	0.147	
(8)	***Three*** (depression, anxiety, stress)	128	51	<0.001	0.876	0.839	**0.070**	0.139	

*x*^2^ = Chi-square. *df* = degrees of freedom. *p* = *p*-value. CFI = Comparative Fit Index. TLI = Tucker–Lewis Index. SRMR = Standardised Root Mean Square Residual. RMSEA = root mean squared error of approximation. ω = McDonald’s ω. DASS = Depression Anxiety Stress Scales. ^1^ 21-item DASS (Antony et al., 1998) [16]. ^2^ 12-item DASS (Ali et al., 2021) [27]; (Osman et al., 2012) [26]. ^3^ 9-item DASS version 1 (Yusoff, 2013) [25]. ^4^ 9-item DASS version 2 (Jacobsen et al., 2024) [28]. ^5^ 12-item Mini-DASS (Monteiro et al., 2023) [24]. ^6^ A modified Mini-DASS version derived from the results presented by Monteiro et al. (2023) [24]. ^7^ Did not converge (reach a stable solution). CFA model fit was assessed using the following criteria (Hu and Bentler, 1998) [33], i.e., models with chi-square *p*-values greater than 0.05, CFI and TLI values greater than 0.95, SRMR values less than 0.08, and RMSEA values less than 0.06 indicate good model fit; CFI values between 0.90 and 0.95 in combination with SRMR less than 0.08 indicate adequate model fit. Bold fit indices indicate good model fit. Fit indices in italics indicate adequate fit.

**Table 3:** Spearman’s rho cross-correlations between DASS subscale scores in a community sample of third-trimester pregnant Swedish women (*N* = 78).

	1	2	3	4	5	6	7	8	9	10	11	12	13	14	15
1 DASS-21 depression	-														
2 DASS-21 anxiety	0.49 **	-													
3 DASS-21 stress	0.76 **	0.47 **	-												
4 DASS-12 depression	0.90 **	0.43 **	0.65 **	-											
5 DASS-12 anxiety	0.33 *	0.93 **	0.37 **	0.27 *	-										
6 DASS-12 stress	0.72 **	0.47 **	0.97 **	0.62 **	0.37 **	-									
7 DASS-9 v. 1 depression	0.89 **	0.41 **	0.68 **	0.67 **	0.29 *	0.64 **	-								
8 DASS-9 v. 1 anxiety	0.62 **	0.65 **	0.60 **	0.55 **	0.50 **	0.58 **	0.55 **	-							
9 DASS-9 v. 1 stress	0.69 **	0.40 **	0.90 **	0.56 **	0.34 *	0.83 **	0.65 **	0.49 **	-						
10 DASS-9 v. 2 depression	0.65 **	0.29 *	0.56 **	0.73 **	0.14 *	0.53 **	0.57 **	0.53 **	0.52 **	-					
11 DASS-9 v. 2 anxiety	0.45 **	0.80 **	0.49 **	0.49 **	0.65 **	0.48 **	0.36 **	0.64 **	0.37 *	0.42 **	-				
12 DASS-9 v. 2 stress	0.66 **	0.41 **	0.86 **	0.55 **	0.31 *	0.87 **	0.60 **	0.57 **	0.71 **	0.52 **	0.47 **	-			
13 Mini-DASS depression	0.75 **	0.36 **	0.62 **	0.78 **	0.17 *	0.59 **	0.69 **	0.55 **	0.54 **	0.90 **	0.48 **	0.59 **	-		
14 Mini-DASS anxiety	0.56 **	0.77 **	0.53 **	0.55 **	0.65 **	0.54 **	0.42 **	0.67 **	0.42 **	0.34 *	0.67 **	0.52 **	0.46 **	-	
15 Modified Mini-DASS anxiety	0.58 **	0.74 **	0.56 **	0.58 **	0.58 **	0.57 **	0.46 **	0.65 **	0.45 **	0.42 **	0.70 **	0.56 **	0.50 **	0.91 **	-
16 Mini-DASS stress	0.70 **	0.45 **	0.92 **	0.65 **	0.34 *	0.93 **	0.59 **	0.56 **	0.71 **	0.57 **	0.53 **	0.86 **	0.60 **	0.52 **	0.56 **

DASS = Depression Anxiety Stress Scales. DASS-21 = 21-item DASS (Antony et al., 1998) [16]. DASS-12 = 12-item DASS (Ali et al., 2021) [27]; (Osman et al., 2012) [26]. DASS-9 v. 1 = 9-item DASS version 1 (Yusoff, 2013) [25]. DASS-9 v. 2 = 9-item DASS version 2 (Jacobsen et al., 2024) [28]. Mini-DASS = 12-item Mini-DASS (Monteiro et al., 2023) [24]. Modified Mini-DASS anxiety = A modified Mini-DASS anxiety scale derived from the results presented by Monteiro et al. (2023) [24]. * *p* < 0.01, ** *p* < 0.001.

**Table 4:** Confirmatory factor analysis (CFA) fit indices for the DASS-9 version 1 and the Mini-DASS, including its modified anxiety subscale one-factor models and scale internal reliability values (McDonald’s ω), were analysed with postnatal data from community samples of mothers at infant ages months 1, 6 and 12.

Postnatal Timepoints, Models	Postnatal CFA Model Fit Indices							
	*χ* ^2^	*df*	*p*	CFI	TLI	SRMR	RMSEA	ω
*Postnatal month 1* (*N* = 62)								
**DASS-9 v. 1 Depression**	- ^1^							0.79
**DASS-9 v. 1 Anxiety**	- ^1^							0.83
**DASS-9 v. 1 Stress**	- ^1^							0.73
**Mini-DASS Depression**	6.61	2	0.037	**0.962**	0.887	**0.036**	0.193	0.88
**Mini-DASS Anxiety**	3.35	2	**0.187**	**0.987**	**0.961**	**0.040**	0.105	0.83
**Mini-DASS Stress**	1.84	2	**0.398**	**1.00**	**1.01**	**0.034**	**0.000**	0.76
**Modified Mini-DASS anxiety**	2.64	2	**0.267**	**0.991**	**0.974**	**0.030**	0.072	0.81
*Postnatal month 6* (*N* = 57)								
**DASS-9 v. 1 Depression**	- ^1^							0.74
**DASS-9 v. 1 Anxiety**	- ^1^							0.87
**DASS-9 v. 1 Stress**	- ^1^							0.84
**Mini-DASS Depression**	12.3	2	0.002	*0.917*	0.750	**0.053**	0.306	0.94
**Mini-DASS Anxiety**	0.92	2	**0.632**	**1.00**	**1.05**	**0.019**	**0.000**	0.78
**Mini-DASS Stress**	2.48	2	**0.290**	**0.994**	**0.983**	**0.030**	0.066	0.82
**Modified Mini-DASS anxiety**	2.70	2	**0.259**	**0.989**	**0.968**	**0.033**	0.080	0.80
*Postnatal month 12* (*N* = 36)								
**DASS-9 v. 1 Depression**	- ^1^							0.62
**DASS-9 v. 1 Anxiety**	- ^1^							0.88
**DASS-9 v. 1 Stress**	- ^1^							0.93
**Mini-DASS Depression**	12.5	2	0.002	0.895	0.686	0.065	0.382	0.84
**Mini-DASS Anxiety**	4.46	2	**0.107**	*0.944*	0.832	**0.055**	0.185	0.75
**Mini-DASS Stress**	3.76	2	**0.153**	**0.975**	*0.926*	**0.034**	0.156	0.88
**Modified Mini-DASS anxiety**	3.22	2	**0.200**	**0.983**	*0.949*	**0.042**	0.130	0.82

*x*^2^ = Chi-square. *df* = degrees of freedom. *p* = *p*-value. CFI = Comparative Fit Index. TLI = Tucker–Lewis Index. SRMR = Standardized Root Mean Square Residual. RMSEA = root mean squared error of approximation. ω = McDonald’s ω. DASS = Depression Anxiety Stress Scales. DASS-9 = 9-item DASS version 1 (Yusoff, 2013) [25]. Mini-DASS = 12-item Mini-DASS (Monteiro et al., 2023) [24]. Modified Mini-DASS Anxiety = a modified scale derived from the results presented by Monteiro et al. (2023) [24]. ^1^ Did not converge (reach a stable solution). CFA model fit was assessed using the following criteria (Hu and Bentler, 1998) [33], i.e., models with chi-square *p*-values greater than 0.05, CFI and TLI values greater than 0.95, SRMR values less than 0.08, and RMSEA values less than 0.06 indicate good model fit; CFI values between 0.90 and 0.95 in combination with SRMR less than 0.08 indicate adequate model fit. Bold fit indices indicate good model fit. Fit indices in italics indicate adequate fit.

## Data Availability

The datasets presented in this article are not readily available because the data are part of an ongoing study and due to ethical considerations. Requests to access the datasets should be directed to Sara Lindeberg, sara.lindeberg@med.lu.se.
